# Laparoscopic partial nephrectomy for multilocular cystic renal cell carcinoma: a potential gold standard treatment with excellent perioperative outcomes

**DOI:** 10.1186/1477-7819-12-111

**Published:** 2014-04-23

**Authors:** Ben Xu, Yue Mi, Li-qun Zhou, Jie Jin, Qian Zhang, Guang-fu Chen

**Affiliations:** 1 Department of Urology, Peking University First Hospital and Institute of Urology, Peking University, National Urological Cancer Center, 8 Xishiku Street, Xicheng District, 100034 Beijing, China; 2 Department of Urology, Chinese PLA General Hospital, No. 28 Fuxing Road, 100853 Beijing, China

**Keywords:** Multilocular cystic renal cell carcinoma, Laparoscopic, Partial nephrectomy, Gold standard, Perioperative outcomes

## Abstract

**Background:**

To report on the perioperative outcomes of laparoscopic partial nephrectomy (LPN) for multilocular cystic renal cell carcinoma (MCRCC) and evaluate the feasibility of this minimally invasive technique as a potential gold standard treatment for MCRCC.

**Methods:**

We retrospectively reviewed the database of surgically pathological findings of patients who were diagnosed with MCRCC at Peking University First Hospital and Chinese PLA General Hospital (Beijing, China) between May 2009 and January 2013. A total of 42 patients with an average age of 48.3 years who were treated with LPN were collected. The patients’ perioperative outcomes were reported and analyzed.

**Results:**

All operations were performed successfully without massive hemorrhage or open conversion. None of patients received lymph node dissection or metastasectomy. Two patients required postoperative transfusion with a mean amount of 175 cc packed red blood cells. Only three patients experienced mild postoperative complications. The mean operative time was 2.4 ± 1.2 hours, including the mean warm ischemia time (WIT) of 23.2 ± 5.7 minutes. The mean estimated blood loss was 72.0 ± 49.6 ml. The mean retroperitoneal drainage was 4.4 ± 1.7 days. The mean postoperative hospital stay was 6.1 ± 1.9 days. Pathologically, 40 (95.2%) of the tumors presented as stage pT1abN0M0, while the remaining two (4.8%) presented as stage pT2aN0M0. No recurrences or new lesions occurred in these patients at a mean follow-up time of 30.0 months.

**Conclusions:**

Although the effective option of LPN is not yet the gold standard treatment for conventional renal cell carcinoma, it should be strongly recommended as a potential gold standard treatment for MCRCC due to the benign nature of MCRCC and the excellent perioperative outcomes provided by LPN.

## Background

Multilocular cystic renal cell carcinoma (MCRCC) is a rare renal tumor that was first recognized in 1982 [1], and has a reported incidence of between 1% and 4% of renal cell carcinomas (RCCs) [2,3]. In general, MCRCC is associated with a low nuclear grade and stage and has a favorable prognosis regardless of tumor size. The 2004 World Health Organization (WHO) classification of kidney tumors also noted its diagnostic criteria and categorized MCRCC as a separate entity with good prognosis [4]. Due to the lack of clear radiological criteria and the difficulty in distinguishing it from other types of renal masses, surgical exploration is prompted for MCRCC.

In fact, the reasonable management of MCRCC is controversial and thus needs clarification to avoid unnecessary overtreatment, such as radical nephrectomy (RN) in simple cases. Partial nephrectomy (PN), also known as nephron-sparing surgery (NSS), performed by an open or a laparoscopic approach, might be proven feasible and efficient because MCRCC has been found not to be affected adversely by large tumor size or advanced stage [5]. Recently, growing experience with laparoscopic PN (LPN) for conventional RCC has demonstrated its potential to duplicate the techniques and outcomes of open partial nephrectomy (OPN) [6]. In our opinion, LPN can similarly be generalized to MCRCC, although handling the cystic lesions is a more challenging procedure than in RCC because of the greater potential for inadvertent cyst puncture and tumor cell spillage [7]. Regrettably, there are still no articles reporting on the perioperative outcomes of LPN for the treatment of MCRCC. To the best of our knowledge, we are the first to evaluate the feasibility of this minimally invasive technique and recommend it as a potential gold standard treatment for MCRCC.

## Methods

Approval for this study was granted by the ethics committees of Peking University First Hospital and Chinese PLA General Hospital (Beijing, China). Written informed consent was obtained from all of the patients.

### Patients

**Figure 1:**
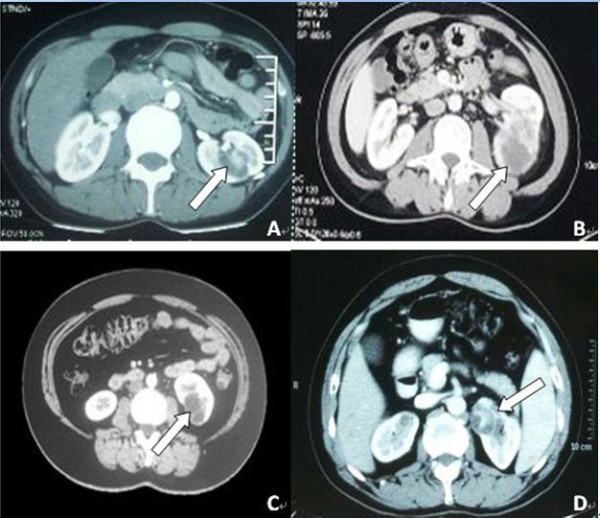
**Preoperative CT scans of the abdomen revealed a typical MCRCC (white arrow). (A)** Typical images of MCRCC localized to the central location of the left kidney. **(B)** Typical images of MCRCC localized to the lower pole of the left kidney. **(C)** Typical images of MCRCC localized to the lower pole of the left kidney. **(D)** Typical images of MCRCC localized to the upper pole of the left kidney. CT, computed tomography; MCRCC, multilocular cystic renal cell carcinoma.

We retrospectively reviewed the database of patients who were diagnosed with MCRCC between May 2009 and January 2013 at Peking University First Hospital and Chinese PLA General Hospital for surgically pathological findings. Patients with bilateral lesions or who had previously undergone renal surgery were excluded from the study. Among them, LPN was performed on 42 patients (33 men and 9 women) with a mean age of 48.3 years (range 32 to 72 years) by two experienced surgeons (QZ and GC). The preoperative evaluations, including urine analysis, serum creatinine (SCr) level, renal B-ultrasonography and computed tomography (CT), were routinely applied to all patients. Preoperatively, both surgeons independently evaluated the CT images, and consultation with at least one radiologist was necessary to improve the accuracy of preoperative diagnosis. After joint discussions, all of the renal masses were confirmed as localized MCRCC without lymph node involvement or distant metastasis. A typical MCRCC is depicted in Figure 1A,B,C,D. Patient demographics, intraoperative variables and postoperative outcomes including follow-up information were reported and analyzed. All of the specimens were examined by at least two experienced pathologists. If the two pathologists disagreed regarding the pathological characteristics, a third specialist was consulted. Clinical follow-up included physical examination, SCr level, chest X-ray and abdominal CT performed at 1 and 6 months and yearly thereafter; this information was obtained from patient charts and referring physicians.

### Surgical treatments

**Figure 2:**
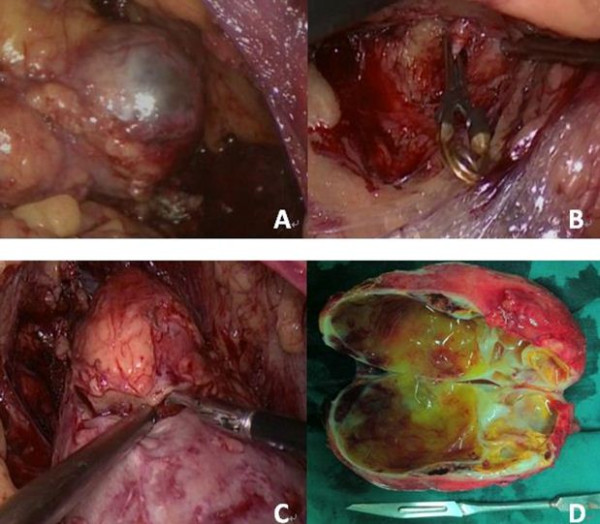
**Intraoperative photographs of LPN for MCRCC. (A)** Laparoscopic photograph of the MCRCC with a clear margin from the normal parenchyma. **(B)** The RA was exposed and clamped completely. **(C)** Laparoscopic photograph of a MCRCC separated from the normal renal parenchyma with cold scissors and the suction device. **(D)** Gross image of resected MCRCC with multilocular cystic lesions. LPN, laparoscopic partial nephrectomy; MCRCC, multilocular cystic renal cell carcinoma; RA, renal artery.

Only three-port, retroperitoneal LPN was performed in all 42 patients. Our surgical technique for treating MCRCC followed essentially the same standard steps for handling other renal tumors, as previously reported [8]. After the construction of a retroperitoneal cavity, the paranephric fat was dissected off Gerota’s fascia, which was subsequently opened and incised away from the tumor (Figure 2A) to facilitate excision and suturing. By dissecting cautiously, the renal vessels were dissected free, and the renal artery (RA) was clamped completely with laparoscopic bulldog clamps (Figure 2B), which was of utmost importance in the treatment of MCRCC. With complete occlusion, the tension of the affected kidney decreased immediately, and the surgeon could obtain a specific tactile sensation to avoid invading the cystic components when dissecting the cystic tumor. After a transient occlusion, the cystic lesion was sharply excised with cold scissors in an almost bloodless field, and the suction device usually accompanied the scissors to aid the separation from the normal kidney (Figure 2C). Afterwards, hemostasis was achieved by suturing any pelvicaliceal entry and using an absorbable oxidized regenerated cellulose bolster. Following renorrhaphy, the vascular clamp was removed, and hemostasis was evaluated. Subsequently, the tumor was retrieved with an endobag, and a suction drain was placed in the retroperitoneal cavity. A representative image of MCRCC is shown in Figure 2D.

## Results

The detailed patient demographics and perioperative outcome characteristics are listed in Table 1. All operations were performed successfully without massive hemorrhage or open conversion. None of the patients received lymph node dissection or metastasectomy. Two patients required postoperative transfusion with a mean amount of 175 cc packed red blood cells. Only three patients experienced mild postoperative complications (two experienced perirenal fluid collection and one experienced excessive drainage), and they were treated successfully by infection control and postponing withdrawal of the drainage tube. The mean operative time was 2.4 ± 1.2 hours (range 1.0 to 4.2 hours), including the mean warm ischemia time (WIT) of 23.2 ± 5.7 minutes (range 14 to 40 minutes). The mean estimated blood loss was 72.0 ± 49.6 ml (range 5 to 200 ml). The mean retroperitoneal drainage was 4.4 ± 1.7 days (range 2 to 7 day). The mean postoperative hospital stay was 6.1 ± 1.9 days (range 3 to 14 days). The SCr levels were all within normal limits, with a preoperative mean of 0.84 mg/dl (range 0.66 to 1.14 mg/dl) and a postoperative mean of 1.01 mg/dl (range 0.62 to 1.48 mg/dl). There was no significant difference (*P* = 0.207, >0.05) between pre- and postoperative SCr levels. Pathologically, all 42 tumors with a negative margin were confirmed to be localized MCRCC (pN0M0) without any lymph node involvement or distant metastasis detected as the previous images had indicated. Among the tumors, 40 (95.2%) presented as stage pT1abN0M0, while the remaining two (4.8%) presented as stage pT2aN0M0. Thirty-five patients (83.3%) were classified into Fuhrman grade 1, and the other seven cases (16.7%) were classified into Fuhrman grade 2. The patients whose follow-up information was available had no evidence of disease at a mean follow-up time of 30.0 months. The cause-specific survival (CSS) rates after undergoing LPN were 100% in the observation periods.

**Table 1:** Patient demographics and perioperative outcome characteristics

**Variable**	**Value**
Number of patients	42
Age (years)	48.3 ± 10.0
Gender	
Male	33 (78.6%)
Female	9 (21.4%)
Tumor location	
Right	24 (57.1%)
Left	18 (42.9%)
Tumor size (cm)	3.4 ± 1.6
Preoperative SCr level (mg/dl)	0.84 ± 0.14
Operative time (hours)	2.4 ± 1.2
WIT (minutes)	23.2 ± 5.7
Estimated blood loss (ml)	72.0 ± 49.6
Drainage days (days)	4.4 ± 1.7
Hospitalization (days)	6.5 ± 2.5
Postoperative SCr level (mg/dl)	1.01 ± 0.19
Stage	
pT1aN0M0	34 (80.9%)
pT1bN0M0	6 (14.3%)
pT2aN0M0	2 (4.8%)
Grade	
Fuhrman 1	35 (83.3%)
Fuhrman 2	7 (16.7%)

SCr, serum creatinine; WIT, warm ischemia time.

## Discussion

MCRCC, defined as an entirely cystic tumor with thin septa containing clusters of Fuhrman grade 1 to 2 clear cells that do not expand the septa [9], is an uncommon histologic subtype (3%) of conventional RCC. Surgical resection can indicate an excellent long-term prognosis for MCRCC, and its biology appears to be more favorable with regards to important prognostic factors such as metastatic presentation, Fuhrman grade, T stage and tumor size [10].

Traditionally, in view of the possibility of tumor rupture or spillage, the suitable treatment for MCRCC is RN. However, at the initial diagnosis, MCRCCs tend to be smaller tumors and have a lower T stage and nuclear grade; therefore, these lesions may be more amenable to PN. Due to accumulated experience with PN and improved surgical techniques, increasing numbers of surgeons have chosen PN as the first therapy for MCRCC in view of its benign nature. Gong *et al*. [11] suggested that an NSS procedure should be considered when a complex multicystic renal mass with enhanced density is observed. In their opinion, rather like conventional RCC, MCRCC is often located in the polar regions of the kidney, making an NSS approach quite feasible. Moreover, You *et al*. [12] indicated that 96% of patients with benign cysts or MCRCCs >4 cm might be able to avoid RN and instead undergo NSS, according to their recent findings. Our clinical data from the present study have also added support to the previous conclusions and, more importantly, have provided recommendations to apply minimally invasive LPN, not OPN, as a potential gold standard treatment, although open nephrectomy remains the gold standard treatment for MCRCC [13].

For more than 4 years, we have been interested in the minimally invasive management of MCRCC with LPN rather than OPN. LPN has been demonstrated as a feasible, efficient and safe technique in stage T1a and even stage T1b conventional RCC, and it seems that LPN can provide similar oncologic results to those of OPN [14]. In our series, all 42 MCRCC cases undergoing LPN experienced successful operations and rehabilitation, although some minor and not severe complications occurred in the early learning curves. These patients not only preserved more renal function with a normal postoperative SCr level but also obtained a good prognosis without any recurrence or metastasis in the follow-up. In fact, LPN has been an acceptable alternative to OPN for the treatment of T1 renal masses [15], although LPN of renal tumors >4 cm still cannot be considered a standard of care [16], and OPN remains the standard of care according to the European Association of Urology guidelines [17]. Some current studies of LPN largely reflect the experience of skilled laparoscopic surgeons at centers of excellence. Nevertheless, the rapid advances in laparoscopic equipment, imaging modalities, renorrhaphy techniques and hemostatic agents have narrowed the proficiency gap to allow LPN to be performed routinely in community settings. Therefore, in view of the fact that LPN has been intensively applied in conventional RCC, mainly in stage T1a conventional RCC, LPN can be considered as an effective therapeutic strategy to treat at least stage T1a MCRCC.

In addition to T1a MCRCC, we believe that larger tumors classified as stage T1b or T2a can also be treated by LPN. In this investigation, we successfully performed LPN in six stage T1bN0M0 cases and in two stage T2aN0M0 cases, in which performing LPN would seem to be contraindicated. However, due to the benign characteristics of these tumors, their size appears to be less important when considering the detailed surgical approach. Some authors have also advocated NSS for renal tumors >7 cm in the presence of a healthy contralateral kidney [18]. Above all, the excellent perioperative outcomes in this study combined with the other related literature have confirmed that LPN is an alternative to open PN or RN for MCRCC. With the development of our experience and skills, LPN has become the gold standard treatment for MCRCC at our institutions.

Since 2011, a new treatment option of ‘zero-ischemia’ LPN has emerged under initially controlled hypotension and later mainly by performing a ‘superselective microdissection’ [19,20]. Although some experts believe that ‘zero-ischemia’ PN could be a safe and effective technique to manage T1 renal tumors regardless of their complexity, the extended operative time and longer learning curve for this complex surgical procedure are obstacles for the majority of urologists to overcome for it to replace conventional LPN as a gold standard treatment for MCRCC. For evaluating the outcomes of PN, Hung *et al*. [21] have introduced a ‘trifecta’ of criteria, which is the combination of a negative cancer margin, minimal renal functional decrease and no urological complications. Based on these criteria, no positive cancer margins or severe urological complications were observed in our series. More importantly, excellent preservation of renal function was also obtained, as shown by the postoperative SCr levels. Given the superior perioperative outcomes with conventional LPN for MCRCC, detractors of ‘zero-ischemia’ LPN continue to raise questions about the real benefits of this procedure when treating MCRCC. In our analysis, only by clamping the hilar vessels to provide a completely bloodless field and a specific tactile sensation to avoid invading the cystic components, can obtaining a negative surgical margin and preserving substantial quantities of renal parenchyma be realized simultaneously; these outcomes cannot yet be easily achieved with a ‘zero-ischemia’ technique. Unlike in conventional RCC, it is especially important in the treatment of MCRCC to avoid invading the cystic components and creating tumor rupture or spillage; in view of the benign characteristics of MCRCC, the ‘zero-ischemia’ technique is not so obvious for MCRCC. We believe that this technique could represent a significant step toward safe and efficient LPN for MCRCC after undergoing the learning curve, but not yet. Advanced ‘zero-ischemia’ skills are strongly required to achieve acceptable results and there is still a long way to go.

Due to the limitations of our study, including its retrospective nature, the fact that it was performed at only two institutions and the limited follow-up, it is still a matter of controversy whether the potential gold standard treatment of LPN for MCRCC can turn into the actual gold standard treatment. Above all, longer and larger prospective studies are still warranted to assess the true advantages and make an accurate long-term assessment of this surgical approach. We look forward to the continuing development of other series to confirm these encouraging results.

## Conclusions

Our results, based on a large series of 42 cases, indicate that LPN is a safe, efficient and minimally invasive therapy for patients with MCRCC. Although the effective option of LPN is not yet the gold standard treatment for conventional RCC, it should be strongly recommended as a potential gold standard treatment for MCRCC due to the benign nature of MCRCC and the excellent perioperative outcomes provided by LPN.

## Abbreviations

CSS: Cause-specific survival; CT: Computed tomography; LPN: Laparoscopic partial nephrectomy; MCRCC: Multilocular cystic renal cell carcinoma; NSS: Nephron-sparing surgery; OPN: Open partial nephrectomy; PN: Partial nephrectomy; RA: Renal artery; RCC: Renal cell carcinoma; RN: Radical nephrectomy; SCr: Serum creatinine; WHO: World Health Organization; WIT: Warm ischemia time.

## Competing interests

The authors declare that they have no competing interests.

## Authors’ contributions

XB and MY carried out the design of this research, analysis and interpretation of data, and drafted the original manuscript. ZLQ and JJ participated in the design of this research and participated in the acquisition of data. ZQ and CGF conceived the study, participated in the operation, reviewed all of the statistical analysis of the data, and revised the manuscript. All authors read and approved the final manuscript.
